# A *heretical* point of view in masonry structures dynamics

**DOI:** 10.1098/rsos.241148

**Published:** 2025-04-30

**Authors:** Mario Argenziano, Enrico Babilio, Yoshiki Ikeda, Massimiliano Fraldi

**Affiliations:** ^1^ Department of Engineering, University of Palermo, Palermo, Italy; ^2^ Department of Structures for Engineering and Architecture, University of Naples ‘Federico II’, Naples, Italy; ^3^ Disaster Prevention Research Institute, Kyoto University, Kyoto, Japan; ^4^ Département de Physique, LPENS, École Normale Supérieure-PSL, Paris, France

**Keywords:** masonry structures, in-plane behaviour, hyperelastic models, nonlinear mechanics, non-smooth contact dynamics, rigid blocks

## Abstract

Protection from static and dynamic actions is an urgent matter for masonry buildings, which constitute the majority of the world’s architectural heritage. For this reason, the search for best strategies to analyse the mechanical responses of such structures under both dead and seismic loads has been at the centre of a vivid debate within the scientific community for decades. Although many different approaches and computational methods have been proposed in the literature over the years, most of them make reference to no-tension materials, starting from the pioneering work by Heyman in the framework of limit analysis. However, implementing the hypothesis of masonry walls made by rigid blocks interacting through no-tension interfaces often leads to inconsistent results due to possible interpenetrating elements. In dynamic simulations, undesired blocks’ interpenetration forces algorithms to continuously check the compatibility and to eventually stop and restart the analysis with somehow arbitrary initial conditions. By introducing well-established hyperelastic and friction laws at bricks’ interfaces, we propose a *heretical* strategy that overcomes some difficulties of the above-mentioned approaches, recovering physical consistency and avoiding any interpenetrations.

## 1. Introduction

Most historic buildings around the world are constructed of masonry. They include various architectural typologies of different sizes and functions and some date back many centuries. Unfortunately, masonry buildings are significantly prone to earthquake damage, due to several factors as the masonry textures, the characteristics of bricks and joints, the restraining among orthogonal walls and, in addition, the numerous alterations made to their original structural integrity over time. According to ICOMOS and ISCARSAH guidelines [1], buildings belonging to cultural heritage cannot be demolished due to their architectural and historical value. Therefore, improving and retrofitting them to withstand seismic loads is crucial to enhance structural safety without altering the architectural character and shape. Any change should be reversible and compatible with the original work. Traditional retrofit interventions may not meet these principles and innovative non-invasive methods should be considered. Within this framework, understanding the mechanics of masonry structures and providing refined models to faithfully describe and predict their complex structural behaviour can open novel perspectives into the field of seismic retrofit applications. In recent years, several methods have gained increasing significance. However, the distinctive characteristics and behaviour of masonry structures and their unique failure mechanisms make accurate modelling quite challenging [2–7]. From a very general point of view, the masonry structure is actually a ‘brick-mortar’ composite structure consisting of hard phase (the brick) and soft one (the mortar). Interestingly from a modelling standpoint, this is analogous to the microstructure in biomaterials [8–10].

Literature evidence shows that the modelling of masonry structures can be generally categorized into three main groups: models treating masonry panels as homogenized continua, models considering the discrete nature of masonry assemblies and limit analysis-based models.

Within the first group, which is the continuum-based models, the finite element method (FEM) is a popular choice because it can handle complex geometries and provide reasonable predictions for full-scale masonry structures [11–19]. However, accurately modelling the behaviour of masonry units and mortar beds remains an open issue. Various approaches have been developed to represent masonry materials, including elastic models and more advanced ones taking into account fracture, residual stresses and progressive damage. Additionally, the type of finite element used (e.g. equivalent-frame elements, brick elements, shell elements or interface elements) and the definition of constitutive models (e.g. linear elastic, nonlinear elastic and damage-plasticity) are crucial for faithfully reproducing the masonry behaviour. Verification and validation of finite element models are essential to ensure accuracy and reliability.

Models in the second group operate from a different perspective. In this regard, a commonly used strategy is to employ discrete element methods (DEMs), which offer advantages such as capturing the discrete nature of masonry units and simulating progressive failure. There are two main discrete element formulations: the distinct element (DE) method [20–26] and the non-smooth contact dynamics (NSCD) [27–33]. These two approaches differ in three main aspects [34]: (i) the contact laws, such as the Signorini’s interpenetration condition and the Coulomb friction relation are regularized through a penalty correction in the DE method, while they are employed, in their original non-smooth formulation in the NSCD strategy; (ii) the DE adopts an explicit integration scheme, and the NSCD method operates through implicit integration; (iii) the DE method considers structural dissipation by assuming Rayleigh damping, while the NSCD method disregards any structural damping, relying solely on perfectly plastic impacts between blocks and friction for dissipation.

Among DE methods, the applied element method (AEM) should be definitely mentioned. Originally adopted for reinforced concrete constructions, it has also been employed for masonry structures [35–37]. In detail, the AEM models the structure as an assembly of relatively small elements, connected by distributed springs in both normal and tangential directions along the edge faces of the elements. The major advantages of AEM are related to the high accuracy of results with respect to other DEM methods with also relatively reasonable computational times. By adopting this method, highly nonlinear behaviour, i.e. the crack nucleation, propagation, separations and rigid body motions of structural elements including the collapse process can be traced back.

A critical discussion to compare the efficiency of continuous and discontinuous methods has recently been published [38].

The third group of models and methods relies on limit analysis. The application of the limit theorems of plasticity for masonry structures was first proposed by Heyman [39–41] under the assumption that the masonry material has no tensile strength, the compressive strength is infinite and sliding between blocks cannot occur. In addition, the blocks are assumed rigid, though Heyman did not state it explicitly. Based on these assumptions, Heyman stated the Safe Theorem assuring that the existence of internal forces in equilibrium with the external loads guarantees that a masonry structure is stable. However, there are counter-examples showing that the Safe Theorem can also fail [42].

Starting from Heyman’s pioneering research works, several approaches have been provided, for analyzing the stability of masonry vaults [43–47] and for investigating the kinematic analysis of recurring seismic failure modes of masonry structures [48,49]. Nevertheless, it is important to acknowledge that limit analysis-based models is used to somehow predict the collapse multiplier and the collapse mechanism, completely neglecting the dynamics of masonry structures, with no information on the displacement and acceleration histories of the structural response.

It should be stressed that most of these different approaches often employ a no-tension constitutive law in which infinite strength and a linear stress–strain relation for compression are assumed, while both stiffness and strength in the tensile regime vanish [3,39–41,50]. Although in some cases the no-tension hypothesis is successfully adopted in dynamic analysis, additional constraints are required to avoid interpenetration among bricks. This inoculates a computational problem often encountered in the numerical simulations of rigid block dynamics: the need to interrupt the analysis when an interpenetration occurs and to jump to the next time step using initial conditions that represent a—somewhat artificial—perturbation of the system state at the previous time step.

To overcome these drawbacks, we propose a *heretical* point of view completely different from the strategies widely employed in the current state of the art. In particular, at least in the absence of crushing phenomena—which can be incorporated separately introducing a proper failure criterion—by starting from the evidence that high contractions of the interface joints occurring as two adjacent blocks get close and call into play high compressive stresses and thus involve high (elastic) energy levels, we replace the standard no-tension models with hyperelastic mechanical relations for describing the actual behaviour of those interfaces. This led to two effects: (i) penalizing in a natural (mechanically consistent) way the energy associated with high contraction states and in turn (ii) strongly discouraging any tendency of blocks to interpenetrate during the dynamics.

To do this, two constitutive laws, i.e. a neo-Hookean and an *ad hoc* modified no-tension relation, are adopted for bricks’ interfaces, for modelling a wide class of masonry panels, ranging from specimens where joints are fully elastic, such as for rubberized mortar or rubber-like joints, to more conventional mortar beds. In addition, we admit the existence of sliding displacements, which are non-Heymanian, between blocks in contact, and we also employ a velocity-dependent dry friction law with smooth static-dynamic passage to take into account dissipative phenomena.

It should be emphasized that the proposed approach is neither a trick for bypassing a purely numerical issue nor a method whose goal is minimizing computational costs. Therefore, this study is not intended to demonstrate that the provided model gives rise to more realistic outcomes in masonry structures analyses when compared with other strategies, but rather it aims to highlight the role and the implications of physically consistent models of interfaces in the global dynamics of a masonry panel. To focus on this very specific aspect, the model is deliberately simplified, under very restrictive assumptions, and some issues are left out. For example, in the current stage of the present research, the model is not equipped with degrees of freedom that allow it to exhibit out-of-plane behaviour. Although such a point is technically highly relevant, and as such we leave it for further developments, introducing it would increase the model complexity, somehow shading the fundamental role of the interface. Indeed, we believe that an in-depth understanding of the dynamics of masonry structures is an essential condition for assisting engineers and architects in design and retrofit applications. It could be further helpful in conceiving new engineered smart interfaces that can enhance the seismic behaviour of masonry panels, by dissipating energy via some mechanisms, e.g. the contact friction between couples of bricks or the cumulated damage in elastic rubber-like joints.

The paper is organized as follows: the introduction of hyperelastic nonlinear constitutive laws for dealing with masonry panels, i.e. the neo-Hookean elastic and the modified no-tension relation, is motivated in §2; numerical tests are implemented and their results are shown for some particular limit cases in §3.

## 2. Problem formulation and modelling strategy

**Figure 1.**
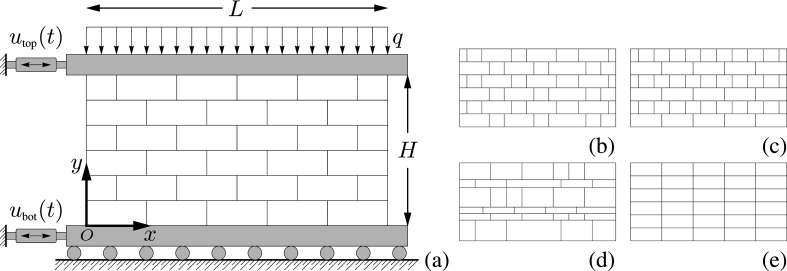
Some views of generic brickwork assemblies: (a) schematic representation of the model problem inspired by a standard experimental setup with panel mounted in between rigid bars used to transfer static and dynamic actions applied by means of actuators; some possible bricklaying patterns that can be considered are (a) the stretcher bond, (b) the Flemish bond, (c) the English cross or Dutch bond, (d) a disordered assembly and (e) the stack bond.

To investigate the in-plane dynamics of brickwork panels, a multi-body system has been considered. The model specimen is a panel of width L, height H and thickness B made of a given number of brick rows in a reference Cartesian frame x,y whose origin O is set in the left bottom corner of the panel. We call x the horizontal axis and y the vertical one. The external actions are the horizontal, time-dependent displacements $u_{\text{bot}}(t)$ and $u_{\text{top}}(t)$ and the vertical, fixed load q, with dimensions of force per unit length. Therefore, the model problem refers to the experimental setup [51], depicted in figure 1a, often employed for studying the dynamical response of panels.

By arbitrarily varying the number of rows, the size of the bricks or the number of bricks per rows, panels with several aspect ratios or characterized by different bricklaying patterns can be considered. For instance, figure 1 shows (a) the stretcher bond, (b) the Flemish bond, (c) the English cross or Dutch bond, (d) a disordered assembly and (e) the stack bond, which is, however, not a structural bond.

**Figure 2.**
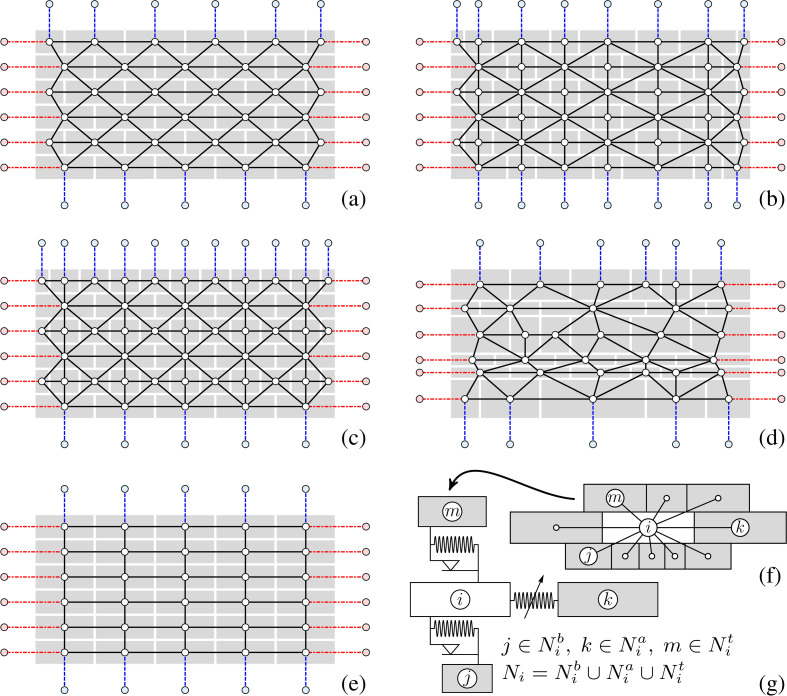
The interaction networks among bricks assembled as (a) stretcher bond, (b) Flemish bond, (c) English cross or Dutch bond, (d) a disordered assembly and (e) stack bond. Black lines: pairwise interactions between bricks. Dashed blue lines: connections among top and bottom brick rows and the corresponding rigid beams. Dot-dashed red lines: possible connections among the specimen and other panels. Bricks are shown as grey-shade rectangles as a reference. (f) The generic brick i and its nearest neighbours (here grey-coloured), in the set $N_{i}.$ (g) A schematic view of the types of interactions among i and its neighbours: linear elastic and dry friction between i and its neighbours in the set $N_{i}^{b}\cup N_{i}^{t}\subset N_{i}$ (see bricks $j\in N_{i}^{b}$ and $m\in N_{i}^{t}$) and nonlinear elastic between i and its neighbours in the set $N_{i}^{a}\subset N_{i}$ (see the brick k).

Interactions among bricks are local and pairwise, ideally implying a network of connections, and are depicted with black-coloured lines connecting the centres of gravity of the bricks in figure 2(a–e), for all the several patterns reported in figure 1. The dashed blue lines represent the connections among the top and bottom brick rows and the corresponding rigid beams. To allow the possibility to connect two or more panels in series or impose external actions to the vertical sides of the wall, the network can be equipped with additional interaction links, shown by dot-dashed red lines in figure 2(a–e).

With reference to a generic inner brick i, we define $N_{i}$ as the set of masses surrounding i and call the mass j, with j≠i, as the nearest neighbour of i if and only if $j\in N_{i}$. Therefore, the masses i and j share part of their boundaries with each other, as shown in figure 2f. Since the wall is built as a superimposition of several rows of bricks, $N_{i}$ is


(2.1) $$\begin{array}{lcr} & N_{i}=N_{i}^{b}\cup N_{i}^{a}\cup N_{i}^{t}\;, & \end{array}$$

where $N_{i}^{b},$$N_{i}^{a}$ and $N_{i}^{t}$ are the sets of nearest neighbours below, adjacent or above the mass i.

The concept of the neighbourhood can be straightforwardly generalized to bricks located on the sides, the bases and the corners of the wall, by taking into account the boundary connections, shown with dashed blue and dot-dashed red lines in figure 2(a–e). Additionally, it should be emphasized that the number of interactions is not *a priori* assigned, but depends on the texture of the considered panel.

Coherently with the reference axes, we call horizontal joints or beds those parallel to the x axis (between two neighbouring masses located at different rows) while vertical joints those parallel to the y axis (between two neighbouring bricks placed at the same line).

### 2.1. Constitutive laws of pairwise interactions

The pairwise interaction laws between masses are detailed below. We refer to figure 2g for notation. In particular, linear elastic forces and dry friction forces are assumed to take place in horizontal joints (shown in figure 2g as j and m, respectively, where $j\in N_{i}^{b}$ or $m\in N_{i}^{t}$), while nonlinear elastic forces develop in the vertical joints (one is reported as an example in figure 2g, connecting the i-th and the k-th brick, being $k\in N_{i}^{a}$).

We highlight that, in existing studies on masonry walls, dynamic dry friction is often considered mostly independent of relative velocity. However, this contradicts experimental evidence [52]. To capture the stick–slip interactions along horizontal joints, we use a specific nonlinear dry friction model that makes the friction force dependent on relative velocity. This is one of the main updates introduced in the mechanical model we are dealing with.

More importantly, the other new aspect is the adoption of interaction laws for vertical joints that have two purposes: they must (i) reproduce the increase in stiffness under severe compressions and (ii) prevent the interpenetration of rigid blocks without additional constraints.

#### 2.1.1. Elastic forces due to horizontal joints

The linear elastic force $F_{i,j}^{\text{le}}=F^{le}(\Delta u_{i,j})$ between the mass i and the mass $j\in N_{i}^{b}\cup N_{i}^{t}$ is dependent on the relative time-dependent displacement $\Delta u_{i,j}=\Delta u_{i,j}(t),$ given by


(2.2) $$\Delta u_{i,j}=u_{i}-u_{j}\;,$$

and satisfies the condition


(2.3) $$F^{le}(\Delta u_{i,j})=-F^{le}(-\Delta u_{i,j})\;,$$

implying that motions from the left to the right and vice-versa (of i with respect to j) have the same energy cost.

**Figure 3.**
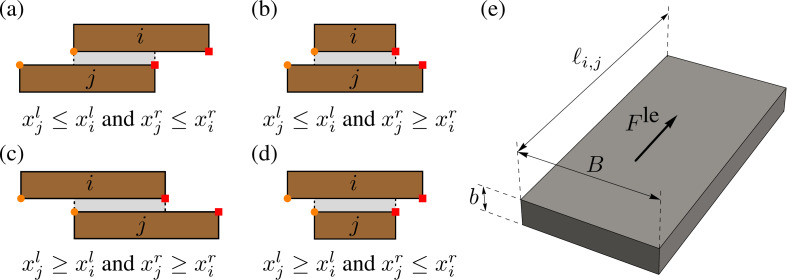
Geometric data for evaluating elastic forces. Panels (a) to (d): set of possibilities of relative positions of the masses i and j to be considered in computing $\ell_{i,j}.$ (e): geometric data of the horizontal bed relevant for the computation of elastic forces. Superscripts l and r refer to the left and the right edge of the brick, respectively. Control points are marked with orange circles and red squares.

The interaction can be written as


(2.4) $$F_{i,j}^{\text{le}}=K_{i,j}\Delta u_{i,j}\;,$$

where the stiffness $K_{i,j}$ is given by


(2.5) $$\begin{array}{lcr} & K_{i,j}=\frac{GB\;\ell_{i,j}}{b}\;, & \end{array}$$

with G the material shear modulus, B the width of the bed (equal to the thickness of the panel), b its thickness and $\ell_{i,j}$ the overlapping length. The latter depends on the relative position of the brick i and j, and is computed by comparing the positions of brick corners, as shown in figure 3.

#### 2.1.2. Elastic forces due to vertical joints

The forces in vertical joints are not required to meet conditions as in equation (2.3). In fact, the brick i approaching or receding $j\in N_{i}^{a}$ is energetically different and governed by pairwise interactions characterized by displacement-dependent stiffness.

From a physical standpoint, we interpret the joints as deformable thin layers and we are firstly interested in modelling in such a way that they constitutively prevent the interpenetration between two adjacent rigid blocks at any time during the dynamic process. In fact, the adoption of artificial conditional expressions that step-by-step correct interpenetration, as often made in numerical simulations in literature, remains strongly unsatisfactory from the mechanical side. Secondly, we require that the stiffness of the attractive response either decreases or vanishes when a block moves away from the other one. To address both these aspects, we model vertical joints as made of an incompressible neo-Hookean material [53–55] undergoing uniaxial extension, for which the strain energy density function is written as


(2.6) $$\begin{array}{lcr} & W=D(\lambda^{2}+2\lambda^{-1}-3)\;, & \end{array}$$

where D is the elastic constant of the material (having the dimension of stress) and λ is the stretch. From equation (2.6), we obtain the stress (in the direction of the extension) as


(2.7) $$\begin{array}{lcr} & \sigma =\frac{\text{d}W}{\text{d}\lambda }=2D(\lambda -\lambda^{-2})\;. & \end{array}$$

**Figure 4.**
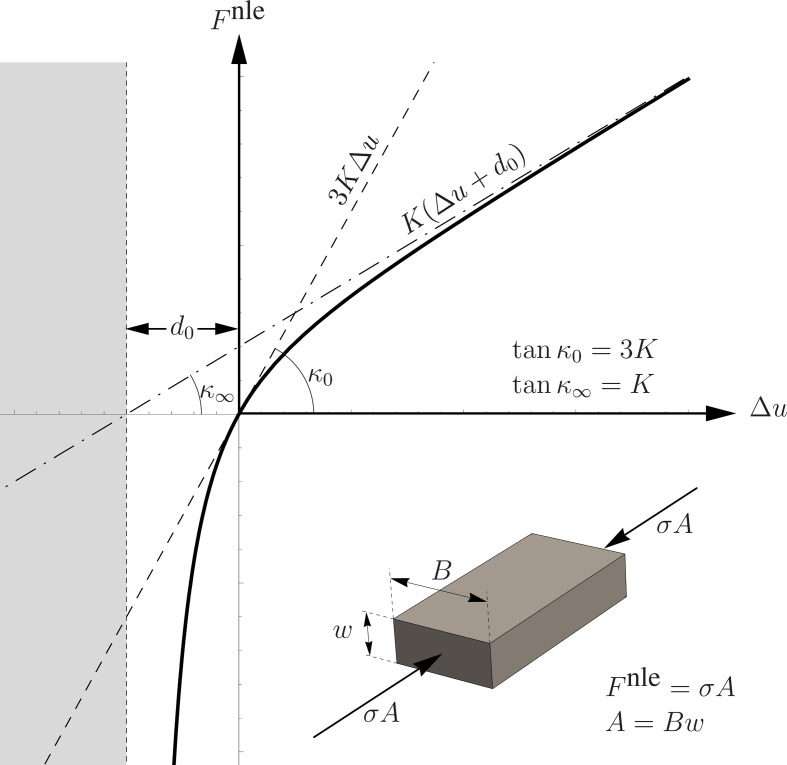
The graph of the neo-Hookean interaction force acting between two adjacent bricks.

With these assumptions, the interaction force between the mass i and its neighbour j is


(2.8) $$F_{i,j}^{\text{nle}}=\sigma_{i,j}A\;,$$

where the area is given by A=Bw,B and w being width and height of the brick (see figure 4).

In order to have zero stress in the reference configuration, we set


(2.9) $$\lambda_{i,j}=\frac{\Delta u_{i,j}+d_{0}}{d_{0}}\;,$$

with $d_{0}$ a distance that we assume is equal to the joint thickness. Therefore, with equation (2.9) put in equation (2.8), the annihilation of such thickness and, consequently, the interpenetration of bricks are penalized.

The asymptotic behaviour of the adopted neo-Hookean relation is such that the limit stiffness K, at Δu→+∞, is one-third of the stiffness $K_{0}$ as the relative displacement approaches 0 (see figure 4). We set that the slope of the force given by equation (2.7) at Δu=0 is equal to that of a linear spring of stiffness


(2.10) $$\begin{array}{lcr} & K_{0}=3K=\frac{EA}{d_{0}}\;, & \end{array}$$

where E is the Young’s modulus of the material of the joint, which we assume is somehow known (i.e. from laboratory tests or from the literature), and therefore the value of D is obtained as


(2.11) $$\begin{array}{lcr} & D=\frac{E}{6}\;. & \end{array}$$

Consequently, the force between two adjacent bricks can be made explicit as


(2.12) $$F_{i,j}^{\text{nle}}=\frac{EA}{3}\left(\frac{\Delta u_{i,j}+d_{0}}{d_{0}}-\frac{d_{0}^{2}}{(\Delta u_{i,j}+d_{0}{)}^{2}}\right)\;,$$

whose graph is shown in figure 4. Notice that equation (2.12) makes sense from the mechanical standpoint in the open interval $\left(-d_{0},+\infty \right)$ while the grey-shaded region shown in figure 4 represents the forbidden interpenetration regime, made energetically not convenient by the neo-Hookean spring. This peculiarity makes the model quite a natural choice, which can also simulate the behaviour of engineered elastic joints or rubber-like beds [56,57]. However, to the best of the authors’ knowledge, using a neo-Hookean law to describe the compression behaviour of vertical joints of masonry panels is unprecedented in scientific literature and far from the conventional and well-accepted methods for masonry structures applications.

**Figure 5.**
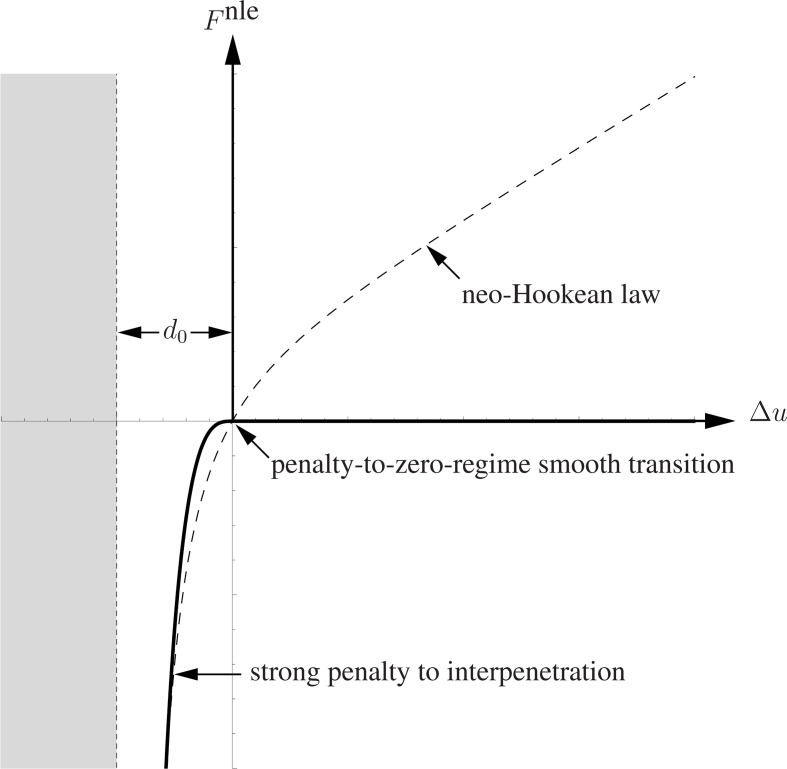
The graph of the modified no-tension interaction force acting between two adjacent bricks.

On the other hand, the model is not able to grasp the no-tension behaviour, nor the brittle or cohesive fracture behaviours usually adopted for masonry assemblies and masonry-like materials, with different levels of accuracy or deepening of physical insight. Therefore, to represent the no-tension behaviour, i.e. the attractive force disappears whenever one block moves away from the other, we replace $F^{\text{nle}}$ defined in equation (2.12) with a new interaction law that still maintains a barrier to prevent interpenetration, such that


(2.13) $$\begin{array}{lcr} & F^{\text{nle}}:(-d_{0},+\infty )\to (-\infty ,\;0\;]\;, & \end{array}$$

whose pairwise analytical description is written as


(2.14) $$\begin{array}{r} F_{i,j}^{\text{nle}}=E^{⋆}A{\left(\frac{\Delta u_{i,j}}{d_{0}}\right)}^{2}\times \left\{ \begin{array}{ll} \tan\left(\frac{\pi }{2}\frac{\Delta u_{i,j}}{d_{0}}\right) & \;\text{if }\Delta u_{i,j}\leq 0\;,\\ 0 & \;\text{otherwise}\;, \end{array}\right. \end{array}$$

being A and $d_{0}$ already defined and $E^{⋆}$ referring to a parameter having dimensions of stress. Notice that the negative branch of equation (2.14) can quantitatively approximate part of the negative neo-Hookean regime for properly tailored values of $E^{⋆}.$ As evident from equation (2.14) and figure 5, the proposed law differs from the no-tension law commonly found in literature, which is characterized by a linear response at negative displacements, without any energy barrier. In addition, equation (2.14) is differentiable at Δu=0 as the neo-Hookean model and unlike the conventional no-tension one. This feature allows for a more plausible representation of physical behaviour, since it may seem unrealistic that materials experience drastic stiffness changes for small oscillations around zero. From this perspective, it can be argued that choosing any hyperelastic nonlinear law with the same properties used here (i.e. no tensile stress, penalty to interpenetration and continuity at zero) would result in a behaviour qualitatively similar to the one described.

To stress the different effects of the described kinds of nonlinear relations, i.e. equation (2.12) or equation (2.14), on the overall in-plane behaviour of masonry walls, some numerical results are reported in §3.

#### 2.1.3. Friction forces

Friction assumes a relevant role in a great variety of processes, including the dynamics of masonry structures. Although its basic properties are common knowledge, in-depth friction details are somewhat elusive. The friction experimentally depends on time and velocity in developing a slide-hold-slide (stick–slip) behaviour. For instance, an increase of static friction is observed upon re-initiation of sliding, followed by a decay to the previous steady-state value, known as the healing effect. In addition, frictional sliding is always accompanied by wear, which is the phenomenon of damage and erosion process of the surfaces. Thus, static friction increases with hold time, while dynamic friction depends on sliding velocity. These observations were fit by an empirical constitutive law by Dieterich [58] and subsequently put into a rate-and-state-dependent friction formulation by Ruina [59]. In the model we consider in what follows, we take into account the dependence of friction on the slip velocity, while neglecting healing and wear effects, since we retain the time duration of the dynamics is quite short. In addition, we assume that friction forces arise only in the direction of the motion of brick rows, that is along the beds between rows, and depends on the compression force orthogonal to the bed.

**Figure 6.**
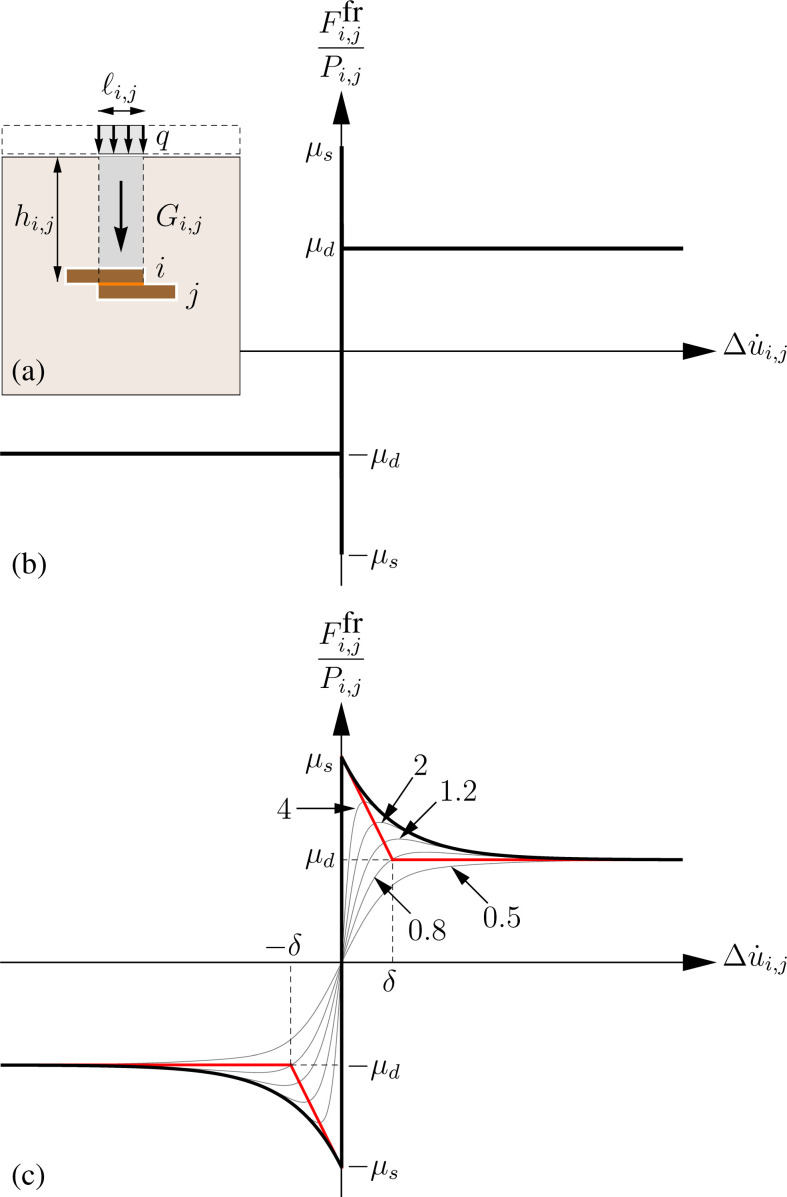
Models for computing the friction force between bricks in static and dynamic phases: (a) the bricks i and j and geometric and mechanical data, (b) graph of the stiction force rule, (c) graph of the adopted velocity dependent rule (black) and its approximated version (grey) for different values of the shape parameter.


With reference to figure 6a, let us consider the brick i, partially overlapping its neighbour j. The compression force is due to the external vertical load $Q_{i,j}$ and gravitational loads $G_{i,j}$ and is computed as

(2.15) $$\begin{array}{lcr} & P_{i,j}=Q_{i,j}+G_{i,j}=(q+\gamma \;h_{i,j}B)\;\ell_{i,j}\;, & \end{array}$$

where γ is the specific weight of the material, B is the thickness of the panel, $h_{i,j}$ is the height of the bed between the bricks i and j from the top of the panel, $\ell_{i,j}$ is the length of the common interface between them (i.e. the overlapping length, the length of the part of the block that covers the one below it) and q is the vertical load per unit length acting at the top of the panel. Note that we are assuming that the materials of bricks and joints have the same γ. Any difference between the bulk densities of the two components is in fact irrelevant since the total volume of joints in the panel is negligible compared to that of the bricks. Here, we consider all the quantities as time-independent.

Defined as $P_{i,j},$ the dynamic friction force $F_{i,j}^{\text{fr}}=F^{fr}(\Delta {\dot{u}}_{i,j})$ between the mass i and the mass j can be assumed dependent on the relative velocity $\Delta {\dot{u}}_{i,j}=\Delta {\dot{u}}_{i,j}(t)$ (i.e. the time derivative of equation (2.2)) given by


(2.16) $$\Delta {\dot{u}}_{i,j}={\dot{u}}_{i}-{\dot{u}}_{j}\;.$$

Here and in what follows, the overdot stands for the derivative with respect to time t. Although more general laws can be chosen, in what follows we assume that the friction force is antisymmetric [60], which implies that the condition


(2.17) $$F^{fr}(\Delta {\dot{u}}_{i,j})=-F^{fr}(-\Delta {\dot{u}}_{i,j})\;$$

is satisfied. Therefore, the motion of the brick i with respect to $j\in N_{i}^{b}\cup N_{i}^{t}$ from the left to the right is penalized in the same way as that from the right to the left.

By choosing the friction force described by the stiction model, we have


(2.18) $$\begin{array}{r} F_{i,j}^{\text{fr}}=P_{i,j}\times \left\{ \begin{array}{ll} {}[-\mu_{s},\mu_{s}]\;,\; & \text{if }\Delta {\dot{u}}_{i,j}=0\\ \mu_{d}\;sign\;\Delta {\dot{u}}_{i,j}\;,\; & \text{if }\Delta {\dot{u}}_{i,j}\neq 0 \end{array}\right.\;, \end{array}$$

where $\mu_{s}$ and $\mu_{d}$ are the static and kinetic friction coefficients, respectively, [.,.] is the multiple-valued function giving all values within the interval of its arguments, and sign(.) is the signum function. Typically, $\mu_{s}\geq \mu_{d}$ holds. The graph of the stiction law is shown in figure 6b.

In several DEM applications on masonry structures of the dedicated literature (e.g. [21,61,62]), as well as in nonlinear dynamic analyses of masses on moving belts [63,64], the Amontons–Coulomb law is frequently used. However, such a special case of equation (2.18), characterized by $\mu_{s}=\mu_{d},$ is not the best choice since it is experimentally observed that $\mu_{s}>\mu_{d},$ and is not able to capture the dependence of the friction force on the relative velocity $\Delta \dot{u}$, known as the Stribeck effect [60,65–68].

To overcome these limitations, we consider the friction force function depicted in the solid black line in figure 6c and defined as


(2.19) $$\begin{array}{r} F_{i,j}^{\text{fr}}=P_{i,j}\times \left\{ \begin{array}{ll} {}[-\mu_{s},\mu_{s}]\;,\; & \text{if }\Delta {\dot{u}}_{i\;,j}=0\\ \left(\mu_{d}+\left(\mu_{s}-\mu_{d}\right)e^{-\left|\Delta v_{i,j}\right|}\right)sign\;\Delta {\dot{u}}_{i,j}\;,\; & \text{if }\Delta {\dot{u}}_{i,j}\neq 0 \end{array}\right.\;, \end{array}$$

where


(2.20) $$\Delta v_{i,j}=\frac{\Delta {\dot{u}}_{i,j}}{\delta }\;,$$

with δ a reference velocity. We emphasize that δ is introduced for dimensional coherence. As shown by the red lines in figure 6c, δ has the geometrical meaning of the abscissa of the intersection point between the tangent lines at $\Delta \dot{u}\to 0^{+}$ and $\Delta \dot{u}\to +\infty .$

However, equation (2.19) is discontinuous, which affects the integration of equations of motion and can imply difficulties when using numerical methods, particularly with variable step-size techniques. In fact, when the function to be integrated is non-smooth, the step-size control of the selected method can lead to undesirable outcomes. These include a reduction in accuracy, due to the lack of discontinuity detection, or a significant reduction in the size of the integration step to meet the tolerance, if the discontinuity has been identified. In the latter case, the process can give rise to two opposite scenarios: either the integration takes an excessively long time and accumulates rounding errors that dominate the solution, or the process halts after reducing the step size to the minimum allowed, failing to achieve the required tolerance.

Therefore, to address these issues due to the lack of smoothness, instead of using equation (2.19), in the following we conveniently take its regularized version defined as


(2.21) $$F_{i,j}^{\text{fr}}=P_{i,j}\left(\mu_{d}+\left(\mu_{s}-\mu_{d}\right)e^{-\Delta v_{i,j}\tanh\left(\phi \;\Delta v_{i,j}\right)}\right)\tanh\left(\phi \;\Delta v_{i,j}\right)\;,$$

where ϕ is a positive dimensionless shape parameter. There exist several regularized friction models, either of public domain available in the literature or specifically defined for commercial multibody simulation packages [69]. The convergence of numerical results obtained using a model or another depends on the set of the adopted parameters. Similarly, one should be aware that the response of the system equipped with equation (2.21) will approximate that one due to equation (2.19) if ϕ is properly chosen. In figure 6c several approximating functions for different values of ϕ, namely ϕ=0.5,0.8,1.2,2 and 4, are shown as grey-coloured curves.

### 2.2. Equations of motion

By considering a system of n bricks and the pairwise interaction forces specified in §2.1, by virtue of D’Alembert’s principle, the equation of motion of the generic brick i is written as


(2.22) $$m_{i}{\ddot{u}}_{i}+\sum_{j\in N_{i}\setminus N_{i}^{a}}\left(F_{i,j}^{\text{le}}+F_{i,j}^{\text{fr}}\right)+\sum_{j\in N_{i}^{a}}F_{i,j}^{\text{nle}}=0\;,\;i=1,\cdots ,n$$

where $m_{i}$ is the mass of the brick and ${\ddot{u}}_{i}$ its acceleration. The sum of forces is computed over the sets of nearest-neighbouring bricks which are below or above the ith brick (i.e. the set $N_{i}\setminus N_{i}^{a}\equiv N_{i}^{b}\cup N_{i}^{t}$) or in the set of those which are adjacent to it (i.e. the set $N_{i}^{a}$), as in figure 2. Notice that, in equation (2.22), the dependence of functions on their arguments is understood for ease of writing.

Initial conditions complementing equation (2.22) are set as


(2.23) $$\begin{array}{lrlr} & & u_{i}=0\;, & \end{array}$$

(2.24) $$\begin{array}{lrlr} & & {\dot{u}}_{i}=0\;, & \end{array}$$

with i=1,⋯,n and the conditions at top and bottom boundaries are


(2.25) $$\begin{array}{lrlr} & & u_{\text{bot}}=U_{\text{bot}}\sin\;\left(\omega_{\text{bot}}\;t\right)\;, & \end{array}$$

(2.26) $$\begin{array}{lrlr} & & u_{\text{top}}=U_{\text{top}}\sin\;\left(\omega_{\text{top}}\;t\right)\;. & \end{array}$$

As shown in figure 2, the proposed model allows considering panels arranged in series by means of the links placed along the left and right panel sides. However, to focus on fundamental issues of the dynamics of a single panel, in the following the lateral links are suppressed and the panel is assumed to be traction-free along its left and right sides and correspondent boundary conditions must not be set.

### 2.3. Dimensionless form of equations

To rewrite equation (2.22) to equation (2.26) in dimensionless form, we introduce the re-scaled displacement $\xi_{i}$ and time τ, respectively, defined as


(2.27) $$\begin{array}{lcr} & \xi_{i}=\frac{u_{i}}{L}\;,\;\tau =\omega t\;, & \end{array}$$

where L is the total length of the panel (see figure 1) and ω the first circular frequency of a reference panel, with the bricks connected only through linear elastic bonds, described by the linear system


(2.28) $$m_{i}{\ddot{u}}_{i}+\sum_{j\in N_{i}\setminus N_{i}^{a}}F_{i,j}^{\text{le}}+\sum_{j\in N_{i}^{a}}K_{0}\;\Delta u_{i,j}=0\;,\;i=1,\cdots ,n$$

with $F_{i,j}^{\text{le}}$ and $K_{0}$ given by equation (2.4) and equation (2.10), respectively.

Taking the largest mass


(2.29) $$\begin{array}{lcr} & M=\max\;m_{i}\;,\;i=1,\cdots \;,n & \end{array}$$

and the largest stiffness of the horizontal joints (see §2.1.1)


(2.30) $$\begin{array}{lcr} & K=\max\;K_{i,j}\;,\;i=1,\cdots \;,n\;\text{and}\;j\in N_{i}\setminus N_{i}^{a}, & \end{array}$$

we introduce the circular frequency


(2.31) $$\begin{array}{lcr} & \Omega =\sqrt{\frac{K}{M}}\;, & \end{array}$$

the re-scaled masses


(2.32) $$m_{i}=\frac{m_{i}}{M}\;,\;0<m_{i}\leq 1\;,$$

and the re-scaled stiffnesses


(2.33) $$K_{i,\;j}=\frac{K_{i,j}}{K}\;,\;0<K_{i,j}\leq 1\;.$$

With these assumptions, equation (2.22) can be rewritten in first-order, dimensionless form as


(2.34)dξidτ=υi,(2.35)dυidτ=−mi−1(Ωω)2(∑j∈Ni∖Nia(Fi,jle+Fi,jfr)+∑j∈NiaFi,jnle),i=1,⋯,n

where


(2.36) $$F_{i,j}^{\text{le}}=\frac{F_{i,j}^{\text{le}}}{K\;L}\;,\;F_{i,j}^{\text{nle}}=\frac{F_{i,j}^{\text{nle}}}{K\;L}\;,\;\text{and}\;F_{i,j}^{\text{fr}}=\frac{F_{i,j}^{\text{fr}}}{K\;L}\;.$$

Similarly, the dimensionless versions of equations (2.23) and (2.24)are written as


(2.37) $$\begin{array}{l} \xi_{i}=0\;, \end{array}$$

(2.38) $$\upsilon_{i}=0,$$

and those of equations (2.25) and (2.26) as


(2.39) $$\begin{array}{r} \begin{array}{rl} & \xi_{\text{bot}}=U_{\text{bot}}\sin\left(w_{\text{bot}}\;\tau \right)\;, \end{array} \end{array}$$

(2.40) $$\begin{array}{r} \begin{array}{rl} & \xi_{\text{top}}=U_{\text{top}}\sin\left(w_{\text{top}}\;\tau \right) \end{array}, \end{array}$$

with


(2.41) $$U_{\text{bot}}=\frac{U_{\text{bot}}}{L}\;\;\text{and}\;U_{\text{top}}=\frac{U_{\text{top}}}{L}\;,$$

(2.42) $$w_{\text{bot}}=\frac{\omega_{\text{bot}}}{\omega }\;\;\text{and}\;w_{\text{top}}=\frac{\omega_{\text{top}}}{\omega }\;.$$

We highlight that the dimensionless form of equations of motion and the corresponding initial and boundary conditions is convenient for numerical purposes. In fact, it allows describing—in a fully parametric way—several panels with different aspect ratios, elastic properties and disordered bricks’ assemblies. In addition, the amplitude and frequency of driving boundary displacements can be *ad hoc* selected as a function of the size and frequency of the reference panel.

## 3. Numerical implementation

The mechanical consistency of the proposed model allows us to capture several cases, ranging from specimens where joints are fully elastic materials (e.g. rubberized mortar or rubber-like joints [56,57]), that quite often are employed for some reparation interventions on existing masonry structures, to cases of dry masonry walls, where mortar joints are absent or even have been pulverized, as a result of stress histories or atmospheric agents.

Additionally, again with reference to the in-plane behaviour of the wall synoptically shown in figure 1, we emphasize that the model can take into account the effects of the aspect ratios of the panel, the dimensions of the masonry bricks and the masonry texture on the overall dynamics of the wall.

It is worth emphasizing that the final goal of this research is not to provide new calculation codes dealing with sophisticated and refined numerical examples, but rather to open a new perspective in which to face the problem of masonry dynamics.

Nevertheless, to integrate equations (2.34)  and (2.35), with pertinent initial and boundary conditions, we hybridize some features of the computer algebra system Mathematica [70] and the programming platform MATLAB [71].

### 3.1. Sketch of the code

We exploit the symbolic capability of Mathematica and numerical capability of Matlab. In particular, Mathematica is used for pre- and post-processing. The former is devoted to building the model geometry equipped with the interaction laws among bricks (see figure 2) and writing of the equations of motion. Then, thanks to the existing routine ToMatlab [72], which has been suitably modified to our scopes, the equations of motion are translated from Mathematica to Matlab language. Through the application software MATLINK [73], properly arranged for the specific problem, the numerical integrations are performed using Matlab, by means of ODE45 or ODE23t routines, depending on the stiffness of the equations. Step size and accuracy are calibrated from test to test. Finally, in the post-processing phase entirely developed in Mathematica, Matlab solutions are analyzed and put in terms of reports and graphics. The step-by-step procedure is summarized in the workflow scheme 1.

### 3.2. Numerical experiments and results

In order to validate the model, several numerical tests were conducted using engineering-relevant parameters for masonry, while only paradigmatic examples are presented here, for brevity. For each one the stiffness of mortar bed is negligible in comparison with that of vertical joints. The latter is characterized by equation (2.12) or by equation (2.14): the first case reproduces masonry panels equipped with rubber-like joints; the second one is for no-tension mortar joints. In both samples, the masonry texture is either regular (stretcher bond) or irregular, as shown in figure 1a,d and the dynamics is activated through a harmonic input at the sole top rigid beam.

**Figure 7.**
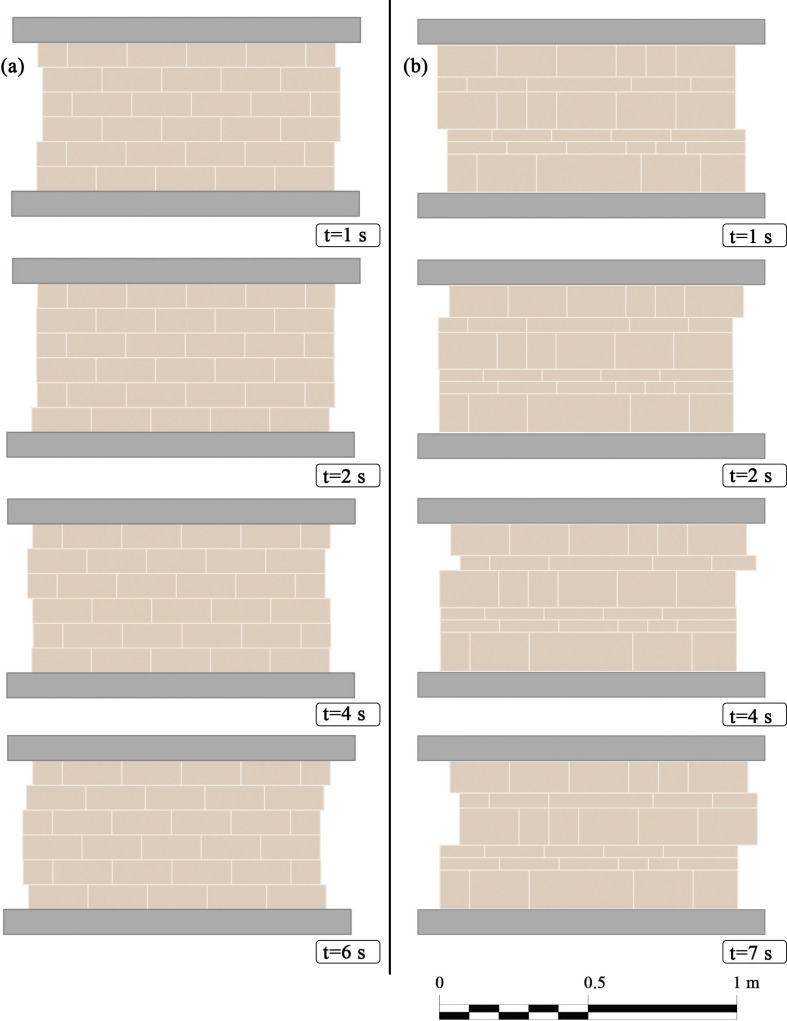
Snapshots of the explicit dynamics of masonry panels: (a) regular and (b) irregular pattern, by adopting the neo-Hookean law for adjacent bricks, whose K_0_ is set to 1.2× 10^7^ kN m^−1^. By assuming a specific weight of 18 kN m^−3^, the length of the panel is 1 m, being the number of rows 6, the aspect ratio 1/2 and the width of the vertical joint equal to 0.01 m for both masonry patterns. The adopted friction parameters are: $\mu_{s}=0.8\;,\mu_{d}=0.3\;,\phi =5.0\;\text{and}\;\delta =0.001\;\text{m}\;{\text{s}}^{-1}$, with a gravity load *q* acting over the panel equal to 500 kN m^−1^. In dynamic simulations, the bottom bar is held steady (*U*_0_ = 0), while the top bar moves with a frequency of 1 Hz and the amplitude *U*_1_ is set to one-tenth of the length of the longest brick, which are respectively 0.20 and 0.35 m for the regular (a) and (b) irregular pattern, i.e. *u*_bot_ = 0 for both cases, being instead u_top_ = 0.02 sin (2πt) and u_top_ = 0.035 sin (2πt) respectively for case (a) and (b).

In detail, figure 7 (whose simulations parameters are reported in the caption) shows deformed shapes of the wall equipped with the neo-Hookean law, i.e. equation (2.12), at selected instants. The two panels, i.e. figure 7a,b, differ only for the masonry pattern, while all other parameters are the same. With some dissimilarities due to the diverse distribution of stiffness that is modulated by the interfaces’ lengths between bricks, as reported in §2.1, one can observe that a sliding behaviour between overlapping rows occurs.

**Figure 8.**
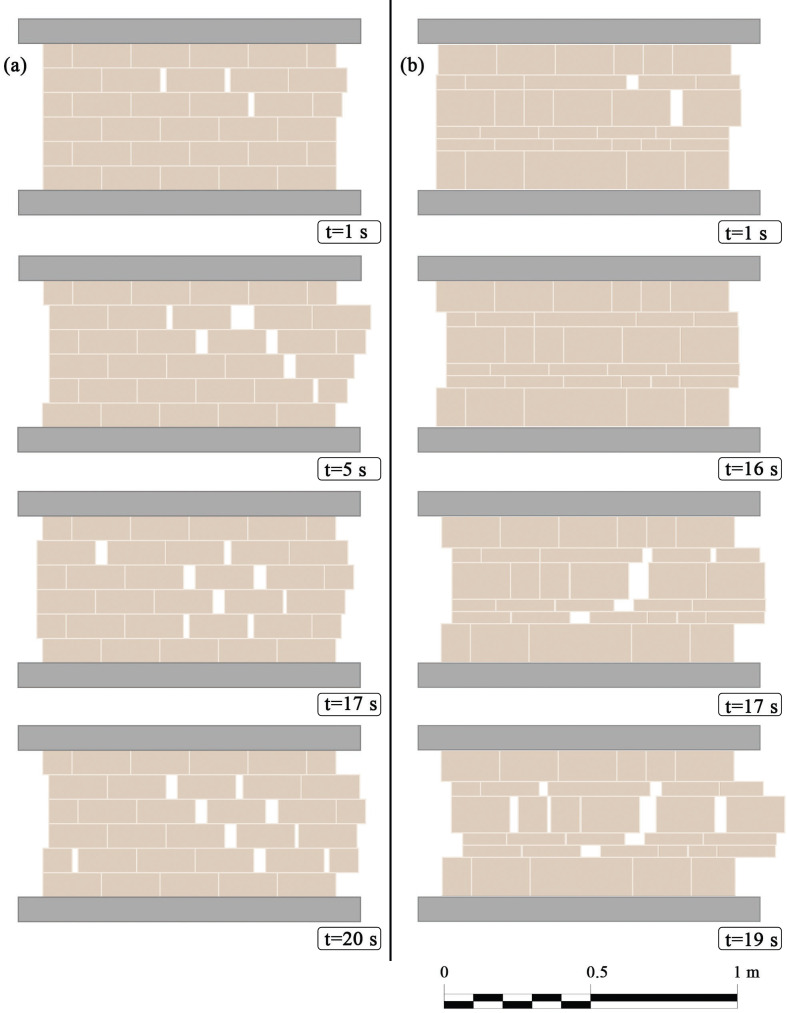
Snapshots of the explicit dynamics of masonry panels: (a) regular and (b) irregular pattern, by considering the proposed no-tension law for adjacent bricks. All the parameters are set as for figure 7 with the sole exception of the frequency of the top bar motion set to 3Hz; in detail $u_{\text{bot}}=0$ for both regular and irregular pattern, while $u_{\text{top}}=0.02\;\;\sin(6\pi t)$ and $u_{\text{top}}=0.035\;\;\sin(6\pi t)$ respectively for case (a) and (b).

Quite different are the results of the second example provided in figure 8. We recall that the no-tension constitutive relation, equation (2.14), replaces the neo-Hookean law, equation (2.12), while the mechanical parameters are the same as in the previous case. The response of both the two masonry patterns is characterized by detachments between adjacent bricks, as a result of the vanishing elastic restoring forces when the tensile regime is experienced by the vertical joints. As one can see by comparing figure 8a,b, the resulting cracking pattern depends on the selected masonry texture.

For both figures 7 and 8, the time instants have been selected in order to report the most significant snapshots that particularize the overall dynamic response of the panels for both regular and irregular patterns.

**Figure 9.**
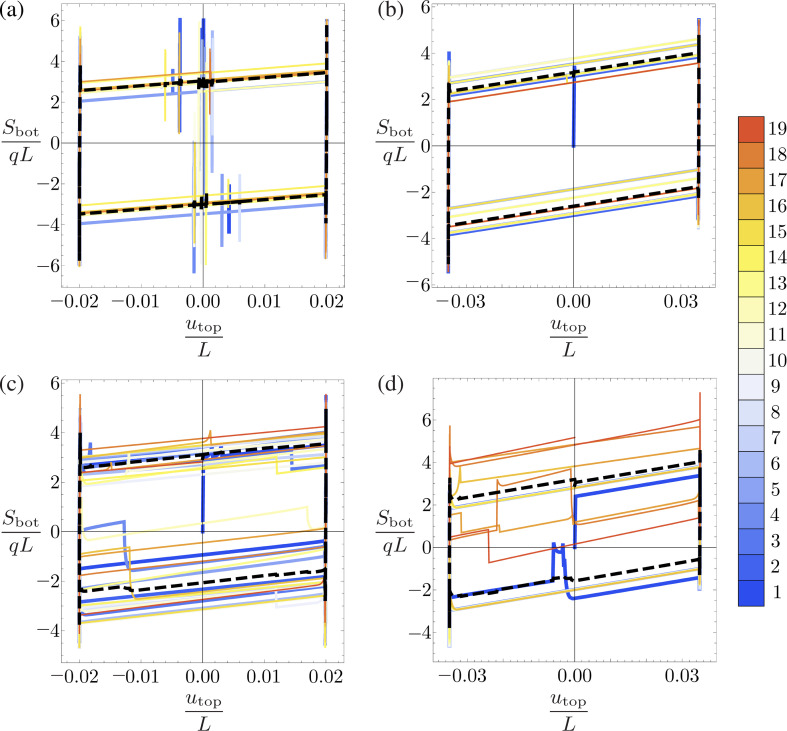
Dimensionless base shear versus dimensionless top displacement: (a) regular brickwork pattern with neo-Hookean bond characterizing vertical joints, (b) irregular brickwork pattern with neo-Hookean bond, (c) regular pattern with no-tension bond characterizing vertical joints, (d) irregular pattern with no-tension bond. Colours in the diagrams allow recognizing at what cycle of the top-bar displacement a given loop corresponds, according to the reported colour code. Black dashed lines refer to the average loop.

To gain further insights into the mechanical behaviour of the masonry panels, in figure 9, which is put in dimensionless form, the base shear force $S_{\text{bot}},$ selected as a performance key parameter, is plot *vs* the driving displacement $u_{\text{top}}$ for the cases displayed in figures 7 and 8.

By comparing the neo-Hookean cases with the no-tension ones, one can notice that: (i) the former are characterized by regular and almost coincident hysteresis loops for both positive and negative displacements; (ii) the latter exhibits an irregular trend strongly dependent on the cycle with different behaviour along the positive and negative displacements.

Additionally, the peaks registered during the base shear histories are due to the combined effects of static and dynamic friction.

It is worth highlighting that the provided base shear plots can be employed as predictive and design tools for estimating the toughness and resilience of real masonry panels for engineering applications. Moreover, the area enclosed by the hysteresis loops is the dissipated energy.

With reference to the computational burdens, it should be emphasized that using no-tension elasticity in a model can lead to instability, thus making it difficult to ensure convergence.
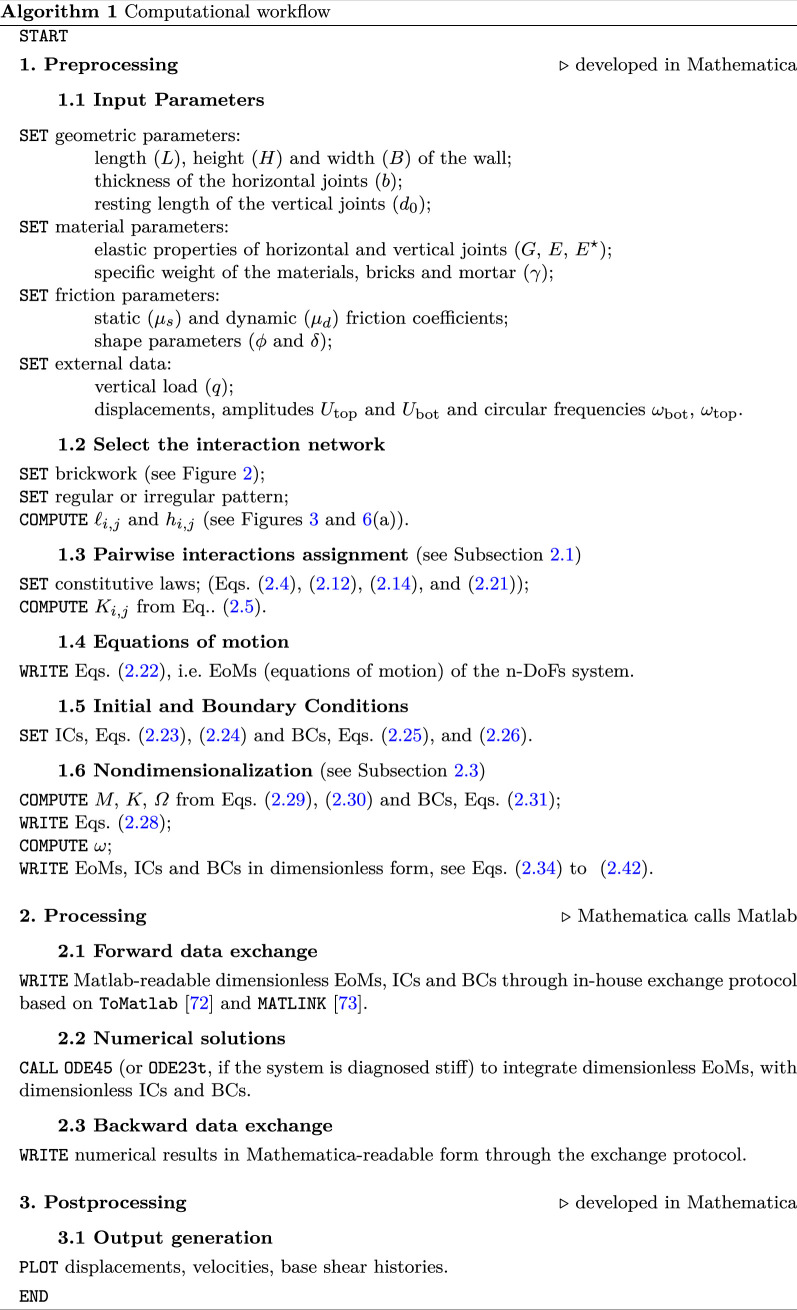


One way to address these difficulties is by superimposing each element utilizing the no-tension link with another element having low elastic moduli values (considerably smaller than those of the element using modified elasticity). This approach introduces a minor ‘artificial’ stiffness, effectively stabilizing the model. Therefore, in the reported results, linear stiffness of joints is taken into account, with shear modulus G and Young’s modulus E set to $1\times 10^{3}\;kN\;m^{-2}$. These values are at least three orders of magnitude less than the other elasticity parameters.

From the numerical results, we found that the mechanical parameters, the static and dynamic friction coefficients and the stick–slip transition velocity are critical to achieving the predicted behaviour, and reducing the number of these key parameters implies impoverishing the model by losing some fundamental information about the response.

Conversely, the shape parameters, e.g. ϕ in the dry friction law or the coefficient 1/2 in the argument of the tangent function of the modified no-tension link, do not qualitatively alter the results. They, at most, introduce slight variations in the displacements and velocity time histories solutions.

Moreover, the values of vertical load acting on the wall and the specific weight of bricks represent threshold parameters that turn on/off the effects of friction on the overall response of the panel, making them non-negligible/negligible.

Finally, it is worth stressing again that, as it can be noticed in examples depicted in figures 7 and 8, the adoption of consistent models, especially in the compression phase, automatically introduces a strong penalty to the interpenetration between blocks, that does not occur.

## 4. Conclusions

With the aim of investigating nucleation and evolution of detachment patterns in assemblies made by linked rigid blocks, we consider a multi-body system composed of single-degree-of-freedom masses moving parallel to each other under external actions imposed on selected boundaries.

Despite the generality of the conceived model, it is here used as a paradigmatic example of a masonry wall subjected to harmonic excitations, where bricks—appearing in arbitrary *opus*—interact with their nearest neighbours through pairwise elastic and friction laws. These reproduce the behaviour of both the horizontal beds and the vertical joints. A variety of mechanical interactions is considered, all functions of the interface lengths or depending on the selected masonry texture.

Although the model is strongly simplified, it points out that combining nonlinear elasticity in compression with smoothed no-tension relation and stick-slip friction gives rise to replicating dynamics in which any tendency of blocks to interpenetrate is strongly penalized by energy levels as growing as the interface is compressed. Indeed, through the provided approach, we show how to avoid—and how to solve in a mechanically consistent way—a classical computational problem one might meet in performing numerical simulations of dynamics of structures made by rigid blocks as, at a given time-step, one or more of those adjacent blocks interpenetrate each other: when it occurs, this forces to first stop calculations for restoring compatibility and then to restart the analysis by imposing somehow arbitrarily new initial conditions, a fact that can lead to obtain user-dependent (or even wrong) results. The issue is compounded when at a given time-step multiple blocks interpenetrate at once, the arbitrariness growing quadratically with the number of relocations to be done for restoring compatible (i.e. non-interpenetrating) brickwork patterns.

In addition, the nonlinear elastic hypothesis is also mechanically consistent with severe contractions eventually experienced by interfaces (i.e. high negative engineering strain or stretches approaching zero).

In conclusion, the *heretical* modelling approach discussed in this work not only overcomes mechanical inconsistencies and numerical issues, but could also be helpful in predicting complex dynamic responses of discrete systems and employed for both designing and retrofitting purposes, as suggested by numerical results. Future efforts will focus on investigating the role of the interaction laws here adopted in more complicated and realistic dynamics, accounting for rocking phenomenon and further degrees of freedom for capturing, for instance, the out-of-plane behaviour of masonry panels.

## Data Availability

The codes and data employed for the research have been provided at Dryad repository [74].
